# ADP-glucose pyrophosphorylase genes are differentially regulated in sugar-dependent or -independent manners in tomato (*Solanum lycopersicum* L.) fruit

**DOI:** 10.5511/plantbiotechnology.23.1004a

**Published:** 2023-12-25

**Authors:** Yong-Gen Yin, Atsuko Sanuki, Yukihisa Goto, Nobuo Suzui, Naoki Kawachi, Chiaki Matsukura

**Affiliations:** 1 Takasaki Institute for Advanced Quantum Science, National Institutes for Quantum Science and Technology (QST), Takasaki, Gunma 370-1292, Japan; 2 Graduate School of Life and Environmental Sciences, University of Tsukuba, Tsukuba, Ibaraki 305-8572, Japan; 3 Institute of Plant and Microbial Biology, Zurich-Basel Plant Science Center, University of Zurich, Zollikerstrasse 107, CH-8008, Zurich, Switzerland;; 4 Tsukuba Plant Innovation Research Center, University of Tsukuba, Tsukuba, Ibaraki 305-8572, Japan

**Keywords:** ADP-glucose pyrophosphorylase, fruit, starch biosynthesis, sugar-signaling, tomato

## Abstract

In early developing tomato (*Solanum lycopersicum* L.) fruit, starch accumulates at high levels and is used by various primary metabolites in ripening fruits. ADP-glucose pyrophosphorylase is responsible for the first key step of starch biosynthesis. Although it has been reported that *AgpL1* and *AgpS1* isoforms are mainly expressed in early developing fruit, their regulatory mechanism has not been elucidated. The present study investigated the transcriptional response of *AgpL1* and *AgpS1* to various metabolizable sugars, nonmetabolizable sugar analogues, hexokinase inhibitors and proline by an experimental system using half-cut fruits. *AgpL1* was upregulated in response to sucrose and constituted hexoses such glucose, whereas the *AgpS1* gene almost did not exhibit a prominent sugar response. Further analyses revealed that other disaccharides such maltose and trehalose did not show a remarkable effect on both *AgpL1* and *AgpS1* expressions. These results indicate that there are two distinct regulatory mechanisms, namely, sugar metabolism-dependent and -independent, for the regulation of AGPase gene expression. Interestingly, the ADP treatment, a hexokinase inhibitors, cancelled the sugar response of *AgpL1*, indicating that hexokinase-mediated sugar signaling should be involved in the sugar response of *AgpL1.* These results suggest that sugar-dependent (*AgpL1*) and sugar-independent (*AgpS1*) pathways coordinatively regulate starch biosynthesis in immature tomato fruit.

## Introduction

Starch is the most important carbohydrate storage substance in plant sink organs. ADP-glucose pyrophosphorylase (AGPase, EC 2.7.7.27) catalyses the first regulatory step of starch biosynthesis by converting glucose-1-phosphate and ATP to ADP-glucose, which is a sole substrate for starch synthase (Lin et al. 1988; Stark et al. 1992). AGPase is allosterically regulated by 3-phosphoglyceric acid (3PGA) and inorganic phosphate (Pi), whose levels control starch biosynthesis (Geigenberger 2011). In higher plants, AGPase is a heterotetrameric enzyme composed of two large subunits (AgpL) responsible for allostatic regulation and two small subunits (AgpS) responsible for catalytic reactions. The presence of both subunits is essential for normal enzymatic function (Okita et al. 1990). In the early developing fruit of tomato, most transported carbohydrates accumulate in plastids as starch (Quinet et al. 2019), and AGPase functions as a rate-determining enzyme in starch biosynthesis (Schaffer and Petreikov 1997; Schaffer et al. 2000).

In tomato, three genes, *AgpL1*, *AgpL2* and *AgpL3*, encoding large subunits, and one gene, *AgpS1*, encoding small subunits have been reported (Chen and Janes 1997; Chen et al. 1998; Park and Chung 1998). It is known that *AgpL* genes are regulated in a tissue- and/or growth stage-specific manner, while *AgpS1* is widely expressed throughout various organs. Regarding *AgpL* genes, major isoforms expressed in early developing fruit, which accumulate starch at high levels, are *AgpL1*, whereas *AgpL2* and *AgpL3* are dominantly expressed in seeds and source leaves, respectively (Chen et al. 1998; Li et al. 2002; Park and Chung 1998; Yin et al. 2010a).

Posttranscriptional regulation of plant AGPase occurs in response to sugar, nitrogen, phosphate and sugar+ABA (Akihiro et al. 2005; Müller-Röber et al. 1990; Nielsen et al. 1998; Scheible et al. 1997; Sokolov et al. 1998). Yin et al. (2010a) reported that salt stress specifically enhances *AgpS1* and *AgpL1* expression in early developing tomato fruit, resulting in higher degrees Brix in red-ripe fruit than under normal conditions. Additionally, it was also reported that transported sucrose specifically upregulates *AgpL1* transcription but not *AgpS1* transcription (Yin et al. 2010a).

In contrast to fruit, Li et al. (2002) reported that *AgpS1*, *AgpL1* and *AgpL2* expression was induced by sugar treatment in leaves. As described here, the expression pattern of AGPase genes is complicated in tomato; however, its regulation has not been fully elucidated, especially in fruit, although the starch accumulation profile is an important factor for its quality under abiotic stress.

To acquire a better understanding of the regulatory mechanism of AGPase expression by sugar and metabolites highly accumulated under salt stress in tomato fruits, the present study established an assay system with half-cut fruits of immature fruits and analysed the responsiveness of *AgpS1* and *AgpL1* genes by treatment with various metabolizable sugars, sugar-analogues, hexokinase inhibitors and proline. The obtained results showed that hexokinase-mediated sugar signaling is involved in the process of sugar-induced expression of the *AgpL1* gene, while sugar-independent signaling is involved in the transcriptional regulation of the *AgpS1* gene in immature tomato fruit.

## Materials and methods

### Plant materials

Seeds of tomato (*Solanum lycopersicum* L., cv. ‘Micro-Tom’) were surface sterilised with 0.6% hypochlorous acid solution for 25 min. After washing with sterilised water three times under sterile bench, the seeds were germinated on moist paper inside a sterilized petri dish in a culture room at 25°C under a light intensity of 110 µmol m^−2^ s^−1^ with a 16 h light/8 h dark photoperiod. When the cotyledons had fully expanded, seedlings were transplanted into rockwool pots (5×5×5 cm) and grown under the same culture conditions by hydroponic cultivation by supplying a commercial nutrient solution (OAT-A prescription; OAT Agrio Co., Ltd., Tokyo, Japan) that was adjusted to an electrical conductivity (EC) of 2.0 dS m^−1^ with distilled water. To investigate AGPase gene expression, immature green fruits at 10 days after pollination (DAP) with 0.5 to 1 cm fruit size were utilised, in which the highest expression level was observed during fruit development (Yin et al. 2010a).

### Treatment of effectors

To investigate the effect of various effectors on AGPase gene expression, a half-cut fruit culture method was established (Supplementary Figure S1). Briefly, 10 DAP fruits were sectioned into two halves at the mid-ring region, and then the cut surface was in contact with the medium after seeds and placenta were removed from the fruits, as shown in Supplementary Figure S1A. The half-cut fruits were placed on a 0.8% agar plate, which contains 1/2 MS salt and the effector(s) described in Supplementary Figure S1B. Then, the fruits were incubated for 6 h at 25°C with a light intensity of 110 µmol m^−2^ s^−1^. Under each condition, 4 fruits (8 halves) from a total of ten plants were subjected. After incubation, the pericarp and columella tissue were stored at −80°C until use for quantitative RT-PCR (qRT-PCR) analyses.

### RNA isolation and cDNA synthesis

Total RNA was extracted from frozen fruit samples with the RNeasy plant Mini kit (QIAGEN, Valencia, CA, USA) according to the manufacturer’s instructions. The extracted RNA was dissolved in RNase-free water and stored at −80°C until use. For cDNA synthesis, 1 µg of total RNA was reverse-transcribed with the PrimeScript First Strand cDNA Synthesis kit (TAKARA BIO Inc., Otsu, Japan) according to the manufacturer’s instructions. The RNA was subjected to reverse transcription with an Oligo-dT^15^ primer.

### Quantitative RT-PCR (qRT-PCR)

Quantitative RT-PCR (qRT-PCR) reactions were carried out with Brilliant SYBR Green QPCR Master Mix (Agilent Technologies, Inc., Santa Clara, CA, USA) by an Mx 3000P qRT-PCR system (Stratagene, San Diego, CA, USA). The expression profiles of the *AgpL1* and *AgpS1* genes were evaluated with gene-specific primers (Yin et al. 2010a). For normalizing the qRT-PCR, the endogenous actin gene *Tom52* (Accession Number. SLU60482) was used as an internal standard (Supplementary Table S1) (Moniz de Sá and Drouin 1996; Yin et al. 2010a). The reaction cycles were as follows: 95°C for 10 min as an initial denaturation, 40 cycles of 95°C for 30 s, 50°C for 30 s and 72°C for 30 s, and 1 cycle of 95°C for 1 min, 50°C for 30 s and 95°C for 30 s (Yin et al. 2010a). Gene expression was calculated in relation to the level of actin gene expression according to the instructions provided by Stratagene based on the method reported by Pfaffi (2001).

## Results

To elucidate the regulatory mechanism of AGPase genes in early-developing fruit, we developed an experimental system using half-cut fruits using 10 DAP immature-green fruits (Supplementary Figure S1), in which the highest expression level was observed (Yin et al. 2010a).

**Figure 1:**
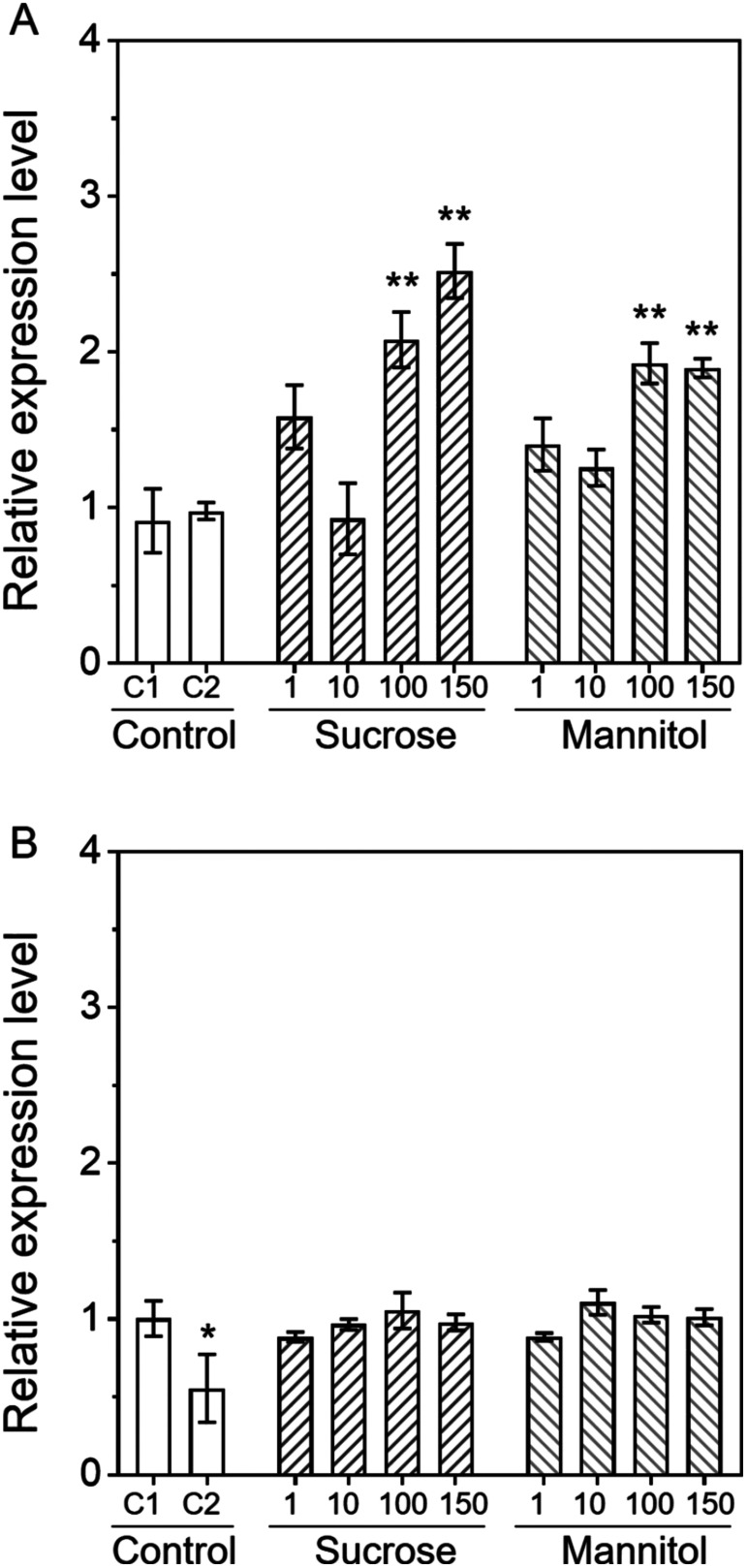
Figure 1. Does-response of *AgpL1* and *AgpS1* expression to sucrose and mannitol in 10 DAP fruit. Half-cut fruits were treated with 1 to 150 mM sucrose or mannitol. C1 (0.8% agar) and C2 (0.8% agar containing 1/2 MS) were used as controls. A and B, Relative transcriptional levels of *AgpL1* and *AgpS1*, respectively. Values are represented by the relative value to the expression level of each gene in C1. The values are means±SEs (*n*=3–4). Asterisks indicate the statistical significance compared to the means of C1 by the one-way ANOVA (* *p*<0.05, ** *p*<0.01).

First, to investigate the dose response to sucrose, the expression levels of *AgpL1* and *AgpS1*, the main isoforms in fruit, were analysed on 1/2 MS medium containing 1 to 150 mM sucrose or mannitol (control for osmotic stress), respectively (Figure 1). Agar plate (Control 1, C1) and 1/2 MS agar plate medium (Control 2, C2) to which nothing was added were defined as controls in this research. *AgpL1* was significantly upregulated by 2.3- and 2.8-fold under 100 mM and 150 mM sucrose, and 2.1-fold under 100 mM and 150 mM mannitol treatments compared to C1, respectively. Although it was upregulated by 1.7-fold under the 1 mM sucrose, and 1.5-fold under the 1 mM mannitol treatments, those were not significant (Figure 1A). On the other hand, *AgpS1* showed no significant change in all treatments compared to the C1 (Figure 1B).

**Figure 2:**
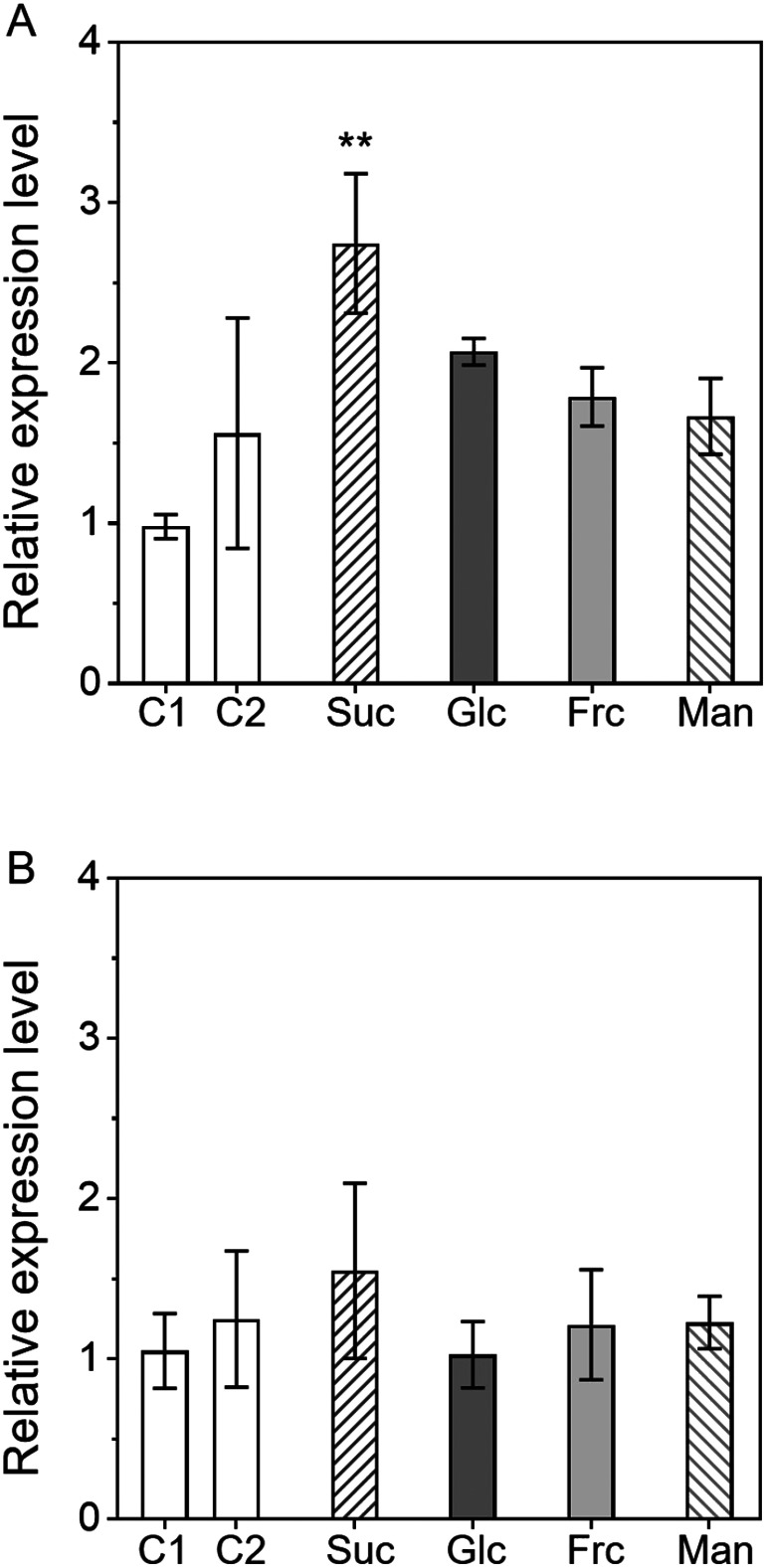
Figure 2. Response of *AgpL1* and *AgpS1* expression to sucrose and its constituent hexoses in 10 DAP fruit. Half-cut fruits were treated with 150 mM sucrose (Suc), glucose (Glc), fructose (Frc) and mannitol (Man). C1 (0.8% agar) was used as a control. A and B, Relative transcriptional levels of *AgpL1* and *AgpS1*, respectively. Values are represented by the relative value to the expression level of each gene in C1. The values are means±SEs (*n*=3–4). Asterisks indicate the statistical significance compared to the means of C1 by the one-way ANOVA (* *p*<0.05, ** *p*<0.01).

Next, we investigated the effects of metabolizable sugars other than sucrose on *AgpL1* and *AgpS1* expression. Figure 2 shows the effect of 150 mM sucrose (Suc), glucose (Glc), and fructose (Frc) on *AgpL1* and *AgpS1* gene expression. *AgpL1* was significantly upregulated 2.8-fold by sucrose (Figure 2A). In addition, even it was not significant, AgpL1 also tended to respond to other metabolizable sugars such glucose and fructose. In contrast, the *AgpS1* gene did not show any significant response to those sugars and sugar alcohols (Figure 2B).

**Figure 3:**
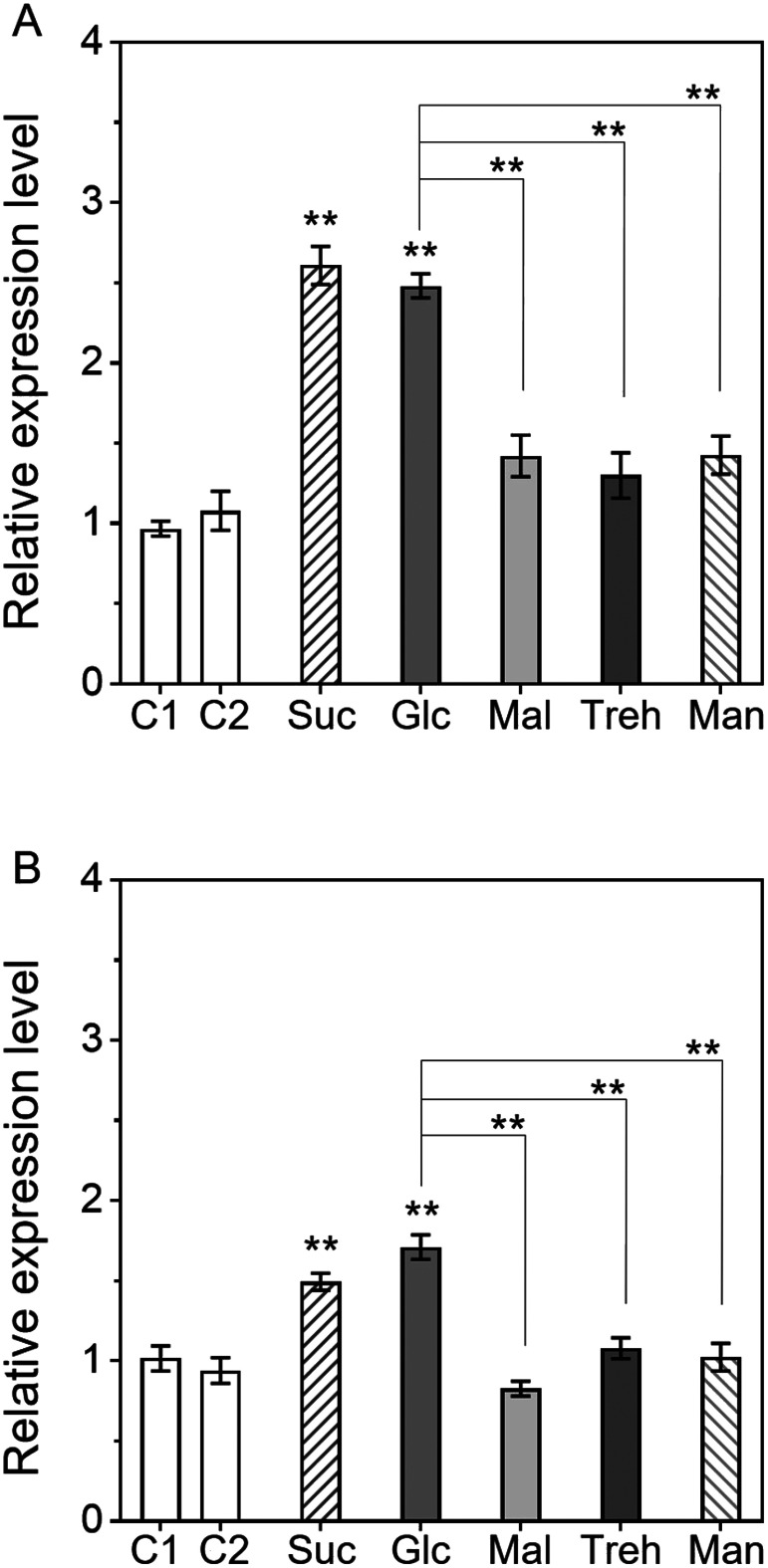
Figure 3. Response of *AgpL1* and *AgpS1* expression to glucose and metabolizable disaccharides in 10 DAP fruit. Half-cut fruits were treated with 150 mM sucrose (Suc), glucose (Glc), maltose (Mal), trehalose (Treh) and mannitol (Man). C1 (0.8% agar) and C2 (0.8% agar containing 1/2 MS) were used as controls. A and B, Relative transcriptional levels of *AgpL1* and *AgpS1*, respectively. Values are represented by the relative value to the expression level of each gene in C1. The values are means±SEs (*n*=3–4). Asterisks indicate the statistical significance compared to the means of C1 or between the mean of the subjects connected by the line by the one-way ANOVA (* *p* <0.05, ** *p*<0.01).

To investigate whether other metabolizable disaccharides influence AGPase expression, the effects of the same concentration (150 mM) of maltose and trehalose on the transcriptional levels of the *AgpL1* and *AgpS1* genes were analysed (Figure 3). While *AgpL1* expression was significantly upregulated by 2.7- and 2.6-fold by sucrose and glucose, respectively, almost no effect of maltose and trehalose was observed (Figure 3A). Although *AgpS1* expression was significantly induced by sucrose and glucose by 1.5-fold and 1.7-fold, its increase level was low compared to that of *AgpL1*. Maltose, trehalose and mannitol did not have significant effects on *AgpS1* expression, similar to *AgpL1* (Figure 3B).

**Figure 4:**
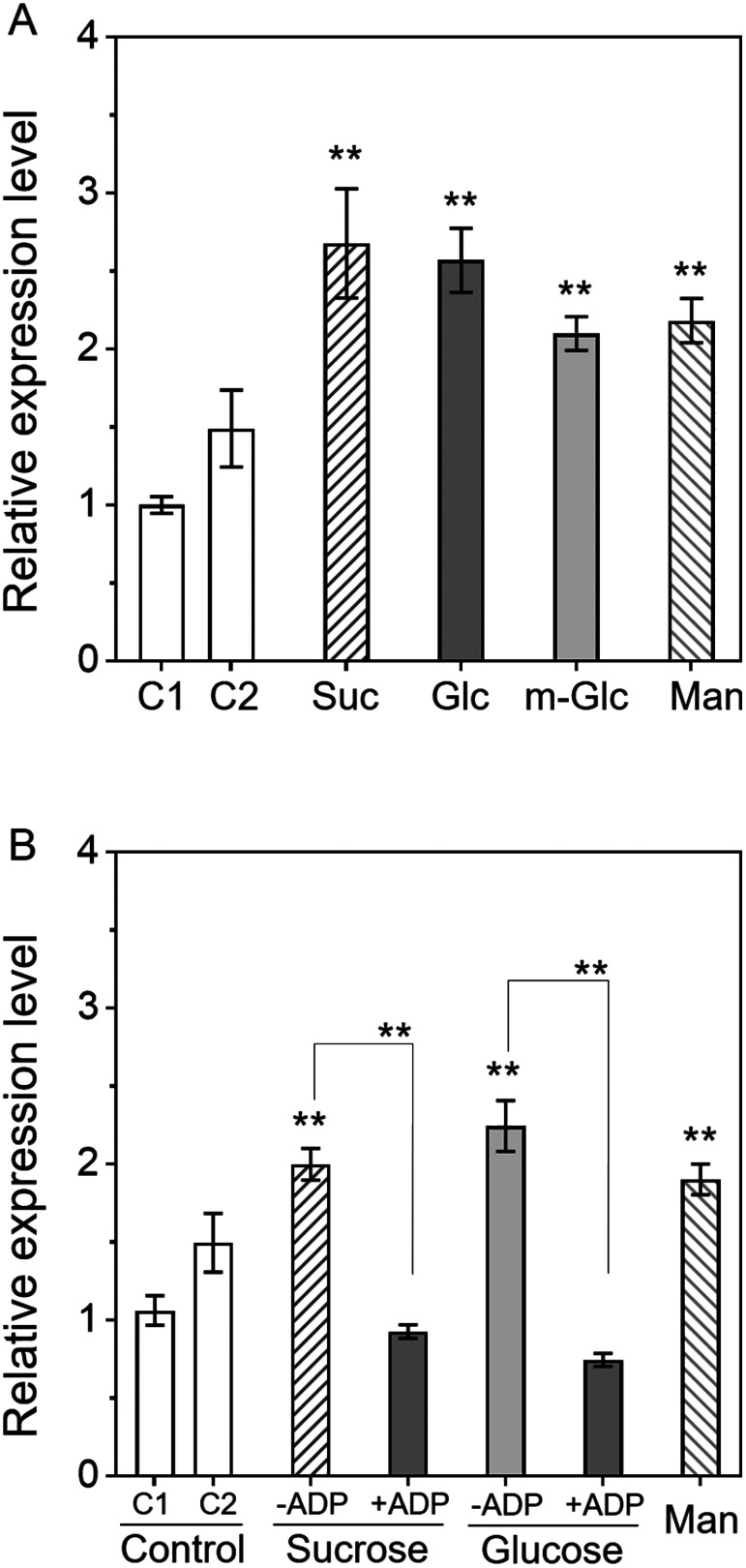
Figure 4. Response of *AgpL1* expression to a nonmetabolizable glucose analogue and hexokinase inhibitor in 10 DAP fruit. A, Half-cut fruits were treated with 90 mM sucrose (Suc), glucose (Glc), 3-*O*-methyl-D-glucose (m-Glc) and mannitol (Man). B, Half-cut fruits were treated with 90 mM sucrose (Suc) and glucose (Glc) with or without ADP and 90 mM mannitol. C1 (0.8% agar) and C2 (0.8% agar containing 1/2 MS) were used as controls. Values are represented by the relative value to the expression level in C1. The values are means ± SEs (*n*=3–4). Asterisks indicate the statistical significance compared to the means of C1 or between the mean of the subjects connected by the line by the one-way ANOVA (* *p* <0.05, ** *p*<0.01).

To further understand the regulation of *AgpL1* expression by sugars, we examined the effect of hexose analogues and hexokinase inhibitors (Figure 4). In these experiments, 3-O-methyl-D-glucose (m-Glc) and adenosine diphosphate (ADP) were used as a hexose analogue and a hexokinase inhibitor, respectively. The treatment conditions, 90 mM Suc, Glc, m-Glc and 5 mM ADP, were as reported by Umemura et al. (1998) and Kandel-Kfir et al. (2006). Consistent with the results in Figures 1 to 3, *AgpL1* was significantly upregulated by 2.7- and 2.6-fold by sucrose and glucose, respectively (Figure 4A). Additionally, in this experiment, significant changes were observed in the m-Glc and mannitol treatments compared to the C1, however, the increase level was lower than the sucrose and glucose treatments (Figure 4A). On the other hand, in Figure 4B, while the sucrose and glucose treatments significantly upregulated *AgpL1* gene expression by 1.9-fold and 2.1-fold, respectively; these responses were cancelled by 5 mM ADP treatment.

**Figure 5:**
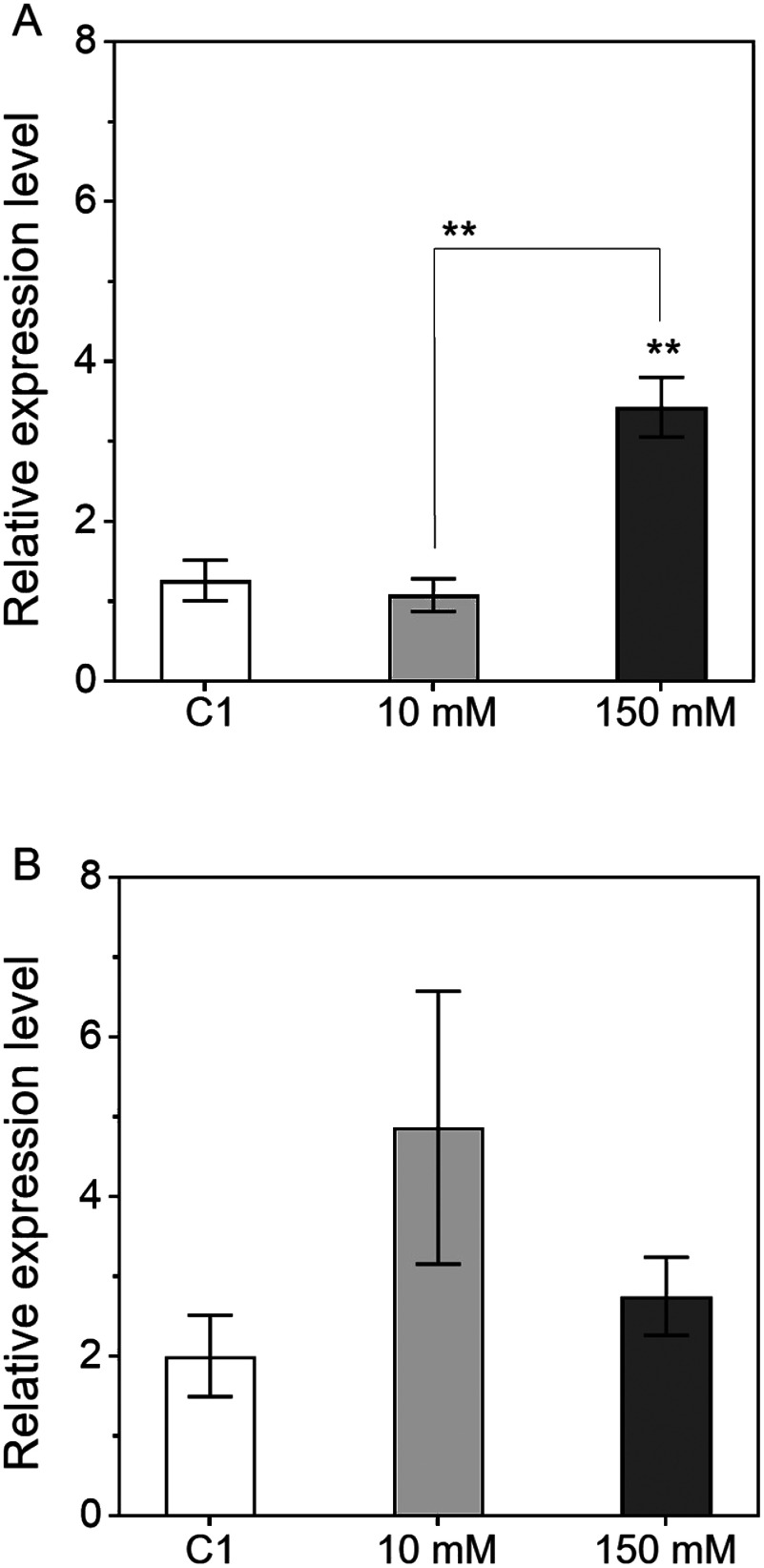
Figure 5. Response of *AgpL1* and *AgpS1* expression to proline in 10 DAP fruit. Half-cut fruits were treated with 10 mM and 150 mM proline. C1 (0.8% agar) was used as a control. A and B, Relative transcriptional levels of *AgpL1* and *AgpS1*, respectively. Values are represented by the relative value to the expression level of each gene in C1. The values are means±SEs (*n*=5–7). Asterisks indicate the statistical significance compared to the means of C1 or between the mean of the subjects connected by the line by the one-way ANOVA (* *p* <0.05, ** *p* <0.01).

Finally, we investigated the effect of proline, which is known to function as an osmoprotectant under salt stress condition, on *AgpL1* and *AgpS1* expression (Figure 5). *AgpL1* expression was significantly upregulated by 2.7-fold only under 150 mM but not 10 mM proline treatment compared to C1. On the other hand, *AgpS1* expression was up-regulated by 2.4-fold under 10 mM proline treatment although not significantly (Figure 5B).

## Discussion

Our previous study reported that *AgpL1* and *AgpS1* genes play important roles in starch synthesis in the early-developing fruit stage, resulting in higher soluble sugar accumulation for red-ripe fruit of tomato under salinity stress (Yin et al. 2010a). In addition, *AgpL1* was upregulated by sucrose in a manner independent of ABA.

In this study, to obtain deeper insight into the mechanism of AGPase gene expression induction by sugar, we examined the effects of various metabolizable sugars, sugar analogues, and hexokinase inhibitors on the expression of *Agp* genes using a half-cut fruit system (Supplementary Figure S1). Treatments with various metabolizable sugars revealed that the *AgpL1* gene responds to sucrose and one of two constituent monosaccharide, glucose at the transcriptional level (Figures 1A, 2A, 3A and 4). On the other hand, regarding the response to mannitol, which is a nonmetabolizable sugar alcohol, an increased expression was observed in Figures 1A, 2A, and 4. Even if its tendency was not stable, a possibility that *AgpL1* responds to osmotic pressure at low levels cannot be excluded as it is also occasionally upregulated in C2 (Figures 2A and 4). However, in any case, the level of expression induction by the metabolizable sugars was greater than that by the mannitol, indicating that the transcriptional induction of *AgpL1* observed in the present study was caused by sugars themselves. These results are consistent with the reports by Li et al. (2002) and Yin et al. (2010a). Interestingly, this upregulation was not observed in other disaccharides, such as maltose and trehalose, although both disaccharides consist of two glucoses (Figure 3A). This result indicates that sucrose and its constituent hexoses, especially glucose, are essential for the sugar response of *AgpL1*. In *Arabidopsis*, it has been reported that *ApL1*, which is considered an orthologue of tomato *AgpL3*, was significantly induced by trehalose in *Arabidopsis* leaves (Wingler et al. 2000). In *Arabidopsis*, it is unclear whether trehalose directly affects *ApL1* expression or after it is decomposed to glucose. In *Arabidopsis*, AGPase genes are known to be differentially responsive to sugar and osmotic pressure; *ApS1*, *ApL2*, and *ApL3* are induced by sucrose and glucose, whereas *ApL1* responds to osmotic pressure (Sokolov et al. 1998). These findings suggest that in tomato fruit, there is a unique mechanism that differs from that in *Arabidopsis* in terms of the sugar response.

The dose response experiment to sucrose indicated that the threshold for the *AgpL1* response to sucrose is between 10 and 100 mM (Figure 1A). Yin et al. (2010a) reported that the sucrose content in the early stage of tomato fruit growth was 5–8 µmol g^–1^ FW (equivalent to approximately 5–8 mM), which is much lower than the threshold suggested above. On the other hand, promoter-GUS transgenic analyses revealed that both *AgpL1* and *AgpS1* are expressed in vascular tissues (Goto et al. 2013; Xing et al. 2005). A similar expression pattern of the AGPase gene was also observed in *Vitis amurensis* (Liang et al. 2022). Verscht et al. (2006) reported that the sucrose content in the perivascular tissue was 50–150 mM in the analysis using castor seedlings. In addition, ^13^C tracer analyses indicated that carbohydrate assimilate transport into fruit mainly occurred in the early developing stage when starch biosynthesis was most active (Yin et al. 2010a). Therefore, it is highly likely that *AgpL1* is regulated at the transcriptional level by sucrose and/or its degraded sugars unloaded from the phloem system in perivascular tissue. To validate how starch biosynthesis is involved in the long-distance assimilate translocation via vascular system and subsequent carbohydrate metabolism, a real-time-imaging analyses for photosynthates such positron-emitting tracer imaging system with starch-defect plant may be effective (Miyoshi et al. 2021; Yin et al. 2021).

In contrast to *AgpL1*, *AgpS1* did not exhibit a consistent response to sugar and mannitol treatments, although it was slightly upregulated by sucrose and glucose, as shown in Figure 3B. These results indicate that there are two pathways for the regulation of AGPase gene expression, sugar-dependent (*AgpL1*) and sugar-independent (*AgpS1*), and both pathways coordinatively regulate starch biosynthesis in immature tomato fruit. It was reported that *AgpS1* expression increases during the early fruit stage and was reinforced by salt stress (Yin et al. 2010a). However, because *AgpS1* did not show any remarkable response to mannitol treatments (Figures 1B–3B), the upregulation of *AgpS1* by salt stress was not due to osmotic pressure. Promoter analyses revealed several *cis*-regulatory elements responsible for the abiotic stress response on the *AgpS1* promoter in a range of crop species, including tomato (Batra *et al.* 2017; Goto et al. 2013). The present analysis revealed the proline induces the expression of *AgpL1* and *AgpS1* (Figure 5). *AgpS1* showed a certain tendency for increased expression by 10 mM proline treatment whereas *AgpL1* did not almost respond to the concentration. Our previous study reported that the proline content in the tomato immature pericarp is usually around 3 µmol g^–1^ FW, but it increases to around 50 µmol g^–1^ FW, more than 16-fold, under 160 mM salt stress condition (Yin et al. 2010b). Taken together, it is likely that *AgpS1* expression is regulated by proline via abiotic stress responses such salinity. However, despite these findings, the regulatory mechanism of *AgpS1* is not fully understood, and further analysis is needed.

**Figure 6:**
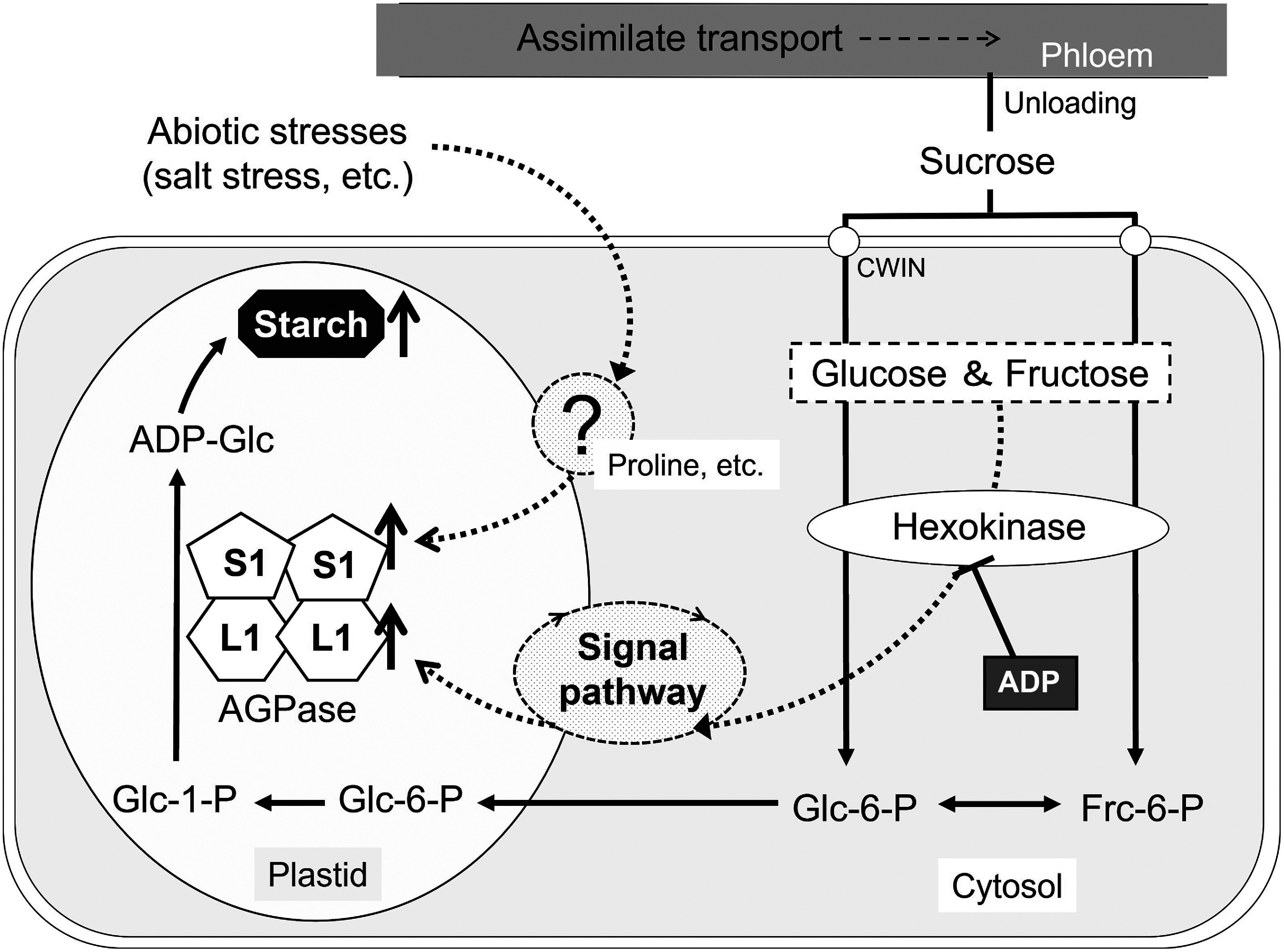
Figure 6. Schematic of the predicted regulatory pathway for AgpL1 and AgpS1 in immature tomato fruit. *AgpL1* is regulated by hexokinase-mediated sugar signaling in the metabolic process of sugars supplied by assimilate transport. In contrast, *AgpS1* is not affected by sugar signals and is regulated by proline induced by abiotic stress, such as salt stress. CWIN, cell wall invertase; Glc-1-P, glucose-1-phosphate; Glc-6-P, glucose-6-phosphate; Frc-6-P, fructose-6-phosphate; ADP-Glc, ADP-glucose.

In this study, we show that *AgpL1* gene expression is upregulated in response to sucrose and only glucose, as sucrose component (Figure 6). Finally, we investigated whether this upregulation is induced by sugar itself or indirectly regulated by the sugar-signaling pathway in the subsequent process of sugar metabolism. To verify these points, the application of sugar analogues and hexose inhibitors was considered effective. Therefore, in this experiment, m-Glc, which is a nonmetabolizable glucose analogue and is not phosphorylated by hexokinase, and ADP, as a hexokinase inhibitor, were applied to half-cut fruit. As a result, *AgpL1* was upregulated by sucrose and glucose with greater levels than by m-Glc. Additionally, those responses to sugars were cancelled by ADP application (Figure 4B). Hexokinase is an enzyme responsible for hexose phosphorylation, which is the initial step for glycolysis. The present results suggested that sugar molecules such sucrose and its constituent hexoses do not directly act as signal molecules in the sugar response of *AgpL1*, but sugar metabolism processes, including hexose phosphorylation, are involved in this response (Figure 6). In higher plants, a number of genes encoding photosynthesis-related enzymes have been reported to be regulated by sugars. Hexokinase has been proposed to function as a sugar sensor in those processes (Sheen et al. 1999). In this study, we demonstrated for the first time that the hexokinase-mediated sugar signaling pathway is involved in the regulation of *AgpL1* expression (Figure 6). Many berry-type fruits are known to accumulate starch in early developing fruit (Roch et al. 2019). It suggests that starch biosynthesis during early fruit development is regulated by sugar translocation from sources to sinks not only in tomato but also in other fruit crops. To validate whether similar regulatory mechanisms are conserved in other berry-type fruit crops, further analysis will be needed.

## Abbreviations

ADP: adenosine diphosphate
AGPase: ADP-glucose pyrophosphorylase
AgpL: ADP-glucose pyrophosphorylase large subunit
AgpS: ADP-glucose pyrophosphorylase small subunit
DAP: days after pollination
Frc: fructose
Glc: glucose
m-Glc: 3-*O*-methyl-D-glucose
qRT-PCR: quantitative RT-PCR
Suc: sucrose

## Description of Supplementary Files

Supplementary Table S1. Primer sequences used for qRT-PCR

Supplementary Figure S1. Treatments of various effectors using half-cut fruit
