# Supplementary material for: ADP-glucose pyrophosphorylase genes are differentially regulated in sugar-dependent or -independent manners in tomato (Solanum lycopersicum L.) fruit
Source: Plant Biotechnol (Tokyo). 2023 Dec 25;40(4):345–51. doi: 10.5511/plantbiotechnology.23.1004a (PMC10905566; doi:10.5511/plantbiotechnology.23.1004a)
Supplement: Supplementary Data [file plantbiotechnology-40-4-23.1004a-s001.pdf]

Supplementary Files

Supplementary Table S1 Primer sequences used for qRT-PCR

Table S1

Gene	Accession No.	Primer (5'-3')	Fragment
AgpL1	U88089	Fw: 5'-GCAGAGAAAGCCACAATTAG-3'	216 bp
		Rev: 5'-ACTTTAGTTTATTTTAGACACGTGTC-3'	
AgpS1	L41126	Fw: 5'-GCTGCTGGCTGCAAAGGG-3'	258 bp
		Rev: 5'-CAAAATCTTGGAGGGCAACC-3'	
Actin	SLU60482	Fw: 5'-CACCATTGGGTCTGAGCGAT-3'	251 bp
		Rev: 5'-GGGCGACAACCTTGATCTTC-3'	

A

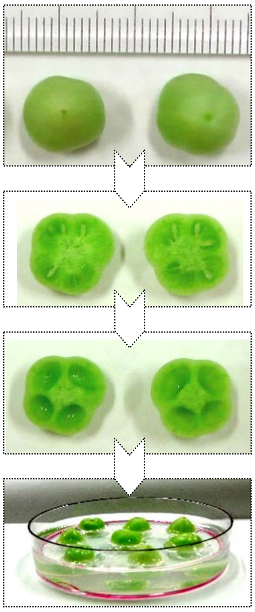

B

Effectors	Fig.1	Fig.2	Fig.3	Fig.4	Fig.5
Control 1 (C1)	0.8% Agar	0.8% Agar	0.8% Agar	0.8% Agar	0.8% Agar
Control 2 (C2)	1/2 MS	1/2 MS	1/2 MS	1/2 MS	-
Sucrose (Suc)	1-150 mM	150 mM	150 mM	90 mM	-
Mannitol (Man)	1-150 mM	150 mM	150 mM	90 mM	-
Glucose (Glc)	-	150 mM	150 mM	90 mM	-
Fructose (Frc)	-	150 mM	-	-	-
Maltose (Mal)	-	-	150 mM	-	-
Trehalose (Treh)	-	-	150 mM	-	-
3-*O*-methyl-D-glucose (m-Glc)	-	-	-	90 mM	-
Adenosine diphosphate (ADP)	-	-	-	5 mM	-
Proline	-	-	-	-	10, 150 mM

Supplementary Figure S1 Treatments of various effectors using half-cut fruit

A. Ten DAP fruits were cut in half and placed on 1/2 MS agar plate medium after removing seeds and jelly. B. Effectors, their concentration and combination applied to the medium.
